# That’s Where Our Humanness Lies”: How Families Leverage Social Capital to Access Antenatal Care in Rural, Eastern Zimbabwe

**DOI:** 10.1177/2752535X251395358

**Published:** 2025-11-20

**Authors:** Anthony Shuko Musiwa, Vandna Sinha, Jill Hanley, Morten Skovdal, Mónica Ruiz-Casares

**Affiliations:** 1 Health Research Methods, Evidence, and Impact, 3710 McMaster University, Hamilton, ON, Canada; 2 Centre for Research on Children and Families, 5620 McGill University, Montreal, QC, Canada; 3 School of Social Work, 5620 McGill University, Montreal, QC, Canada; 4 School of Education, 1877 University of Colorado, Boulder, CO, USA; 5 Sherpa University Institute, Montreal, QC, Canada; 6 Department of Public Health, 4321 University of Copenhagen, Copenhagen, Denmark; 7 School of Child & Youth Care, 7984 Toronto Metropolitan University, Toronto, ON, Canada

**Keywords:** social capital, local informal support, antenatal care, *Ubuntu*, Zimbabwe

## Abstract

Families across sub-Saharan Africa (SSA) often rely on social capital to access traditional, faith-based, and biomedical antenatal care (ANC). Yet, many current studies focus on biomedical ANC and emanate predominantly from the Global North. In this qualitative case study, we aimed to explore how families utilize social capital to access various types of ANC in Mafararikwa, a rural ward in Eastern Zimbabwe. We generated data through interviews with 30 health professionals, and consultations, focus group discussions, and storyboarding with 71 community-level key informants and parents or legal guardians. We analyzed the data thematically. We identified four main themes: Theme 1: Support through social networks and community groups (families draw support from social networks, local groups, and community initiatives to engage with different types of ANC); Theme 2: Material and non-material support (families draw material and non-material supports from social networks, local groups, and community initiatives to engage with various forms of ANC); Theme 3: Reciprocal norms and sociocultural practices (families leverage reciprocal actions, norms of respect and courtesy towards pregnant women, mutual trust, and socio-cultural practices to engage with diverse ANC types); and Theme 4: Informal supports and care mixing dynamics (local informal supports shape families’ interactions with different types of ANC). The complex ways families in Mafararikwa leverage social capital to access preferred forms of ANC reflect context-specific relationships and holistic health conceptions grounded in *Ubuntu*. Culturally-specific responses that strengthen these relationships are needed to ensure equitable ANC outcomes in Mafararikwa and beyond.

## Introduction

Despite its importance in improving maternal and child health, research on how families leverage social capital to access antenatal care (ANC) in rural sub-Saharan Africa (SSA) is limited. ANC refers to care provided to pregnant women by a “skilled provider” to ensure the best pregnancy and health outcomes.^1,2^ The term “skilled provider” is in quotation marks to emphasize its contested definition across different contexts. For instance, in biomedical contexts, it includes medical doctors, nurses, and nurse-midwives.^1,3^ In African traditional care systems, it includes traditional midwives and traditional healers.^2,4^ Social capital refers to resources available through interpersonal networks that enable collective action towards mutual goals.^5^ Examples of social capital include social networks, local groups, community organizations, shared norms, and trust.^5^ Much has been documented about social capital in other health fields across SSA, particularly HIV treatment engagement^6,7^ and sexual and reproductive health knowledge and behaviours.^8,9^ Gleaning from existing reviews, only a handful of studies on ANC engagement exist and these predominantly focus on the uptake or use of biomedical or health facility-based ANC.^10,11^ Biomedical ANC refers to forms of care (e.g., ultrasound foetal monitoring, biomedical treatments for malaria or HIV) given to pregnant women based on (originally Euro-Western) medical and biological notions about pregnancy and health.^1,12^

Current research across SSA often overlooks how social capital supports the use of traditional or faith-based ANC whether separately or alongside biomedical ANC—a practice we call *ANC pluralism*.^13^ Traditional ANC refers to pregnancy care practices rooted in African indigenous knowledges and cultures that are largely passed down through generations.^13,14^ It includes traditional midwifery, herbal remedies, spiritual rituals, dietary guidelines, and protective customs.^13,14^ Faith-based ANC refers to religious or spiritually guided pregnancy care practices shaped by beliefs in divine protection, healing, and intervention grounded in religious faiths such as Christianity and Islam.^4,13^ It often includes prayer, fasting, spiritual counseling, and rituals led by religious and faith leaders.^4,13^ Along with biomedical ANC, traditional and faith-based ANC are not exclusive typologies as they often conflict, overlap, and reinforce each other in complex ways.^13^ The use of different ANC types together indicates diverse and holistic experiences of pregnancy, ANC, and health in SSA.^13,15^ Overlooking these issues risks misaligned ANC policies and care models that ignore local realities, thereby reducing effectiveness and cultural relevance.

In this context of ANC pluralism, we examine how families leverage social capital to access ANC in Mafararikwa in rural Eastern Zimbabwe. Our paper contributes original evidence that recognizes and embraces how and why social capital enables families to utilize different forms of ANC simultaneously. Our study reveals that families in Mafararikwa draw material and non-material support from their social networks, local groups, and community initiatives and leverage their reciprocal actions, norms of respect and courtesy towards expectant women, mutual trust, and socio-cultural practices to access different types of ANC. Thus, strengthening local informal support mechanisms can enhance access to ANC and holistic health outcomes in Mafararikwa and other similar rural areas in Zimbabwe and SSA.

## Social Capital and Access to ANC in Rural Zimbabwe

Like many SSA countries, Zimbabwe has substantially expanded coverage of ANC and improved maternal and child health outcomes among its populations over the last four decades,^3^ mainly due to implementing ANC policies using a primary healthcare strategy.^16^ From 1999 to 2019, Zimbabwe’s ANC coverage increased from 64% to 72%, maternal mortality ratio (MMR) fell from 695 to 462 deaths per 100,000 live births, and under-five mortality rate (U5MR) declined from 102 to 65 deaths per 1000 live births.^3,17^ Despite this progress, rural Zimbabwe experiences many more preventable pregnancy-related deaths among women and children compared to urban Zimbabwe.^16,18^ In 2022, Zimbabwe’s rural MMR was estimated as 402 deaths per 100,000 live births (relative to 298 in urban areas), and rural U5MR was noted as 70 deaths per 1000 live births (compared to 30 in urban areas).^18^ Reflected in many SSA countries too,^19,20^ these gaps in mother and child health outcomes in rural Zimbabwe point to ongoing challenges in ANC access that require urgent policy attention.^3,16^

Social capital is recognized in SSA and worldwide as a potent mechanism for facilitating ANC access and improving the health and lives of women, children, and families.^5,10^ Current conceptualizations identify at least two broad dimensions of social capital. First is structural social capital, which includes resources provided through *social networks* (connections formed through interpersonal relationships), *civic participation* (individual or shared actions on issues of public concern), and *political participation* (individual or joint efforts to influence political decisions or others around societal issues).^5,21^ Structural social capital can be measured objectively by analyzing people’s behaviours and actions using such measures as the number of existing civic groups, size of social networks, amount of network resources, and participation or attendance rates in community groups, such as mutual aid associations.^5,21^

The second dimension of social capital is cognitive social capital, which entails trust, reciprocity, and effective norms typically provided through *collective efficacy* (the joint actions of communities or groups towards mutual benefits), *social cohesion* (community or group members’ emotional and social investments that reflect connectedness and solidarity), and *social control* (the ability of communities or groups to regulate their members’ actions towards shared goals).^5,21^ Cognitive social capital can be measured subjectively by examining people’s attitudes or perceptions, for example, through assessing individual or group perceptions of social belonging and integration, trust in others, and attitudes towards helping others.^5,21^

Existing framings also typically conceptualize social capital into three forms: bonding, bridging, and linking.^5,22^ Bonding means support within close-knit groups with shared attributes (e.g., age, ethnicity, class); bridging entails support across networks of diverse backgrounds; and linking refers to trust and respect between people and formal institutions.^5,22^ Each form includes structural and cognitive dimensions.^21,22^ For example, family ties reflect bonding social capital’s structural side, while shared trust reflects its cognitive dimension. Bridging social capital includes cross-group connections (structural) and mutual respect (cognitive), while linking social capital involves institutional access (structural) and trust in those institutions (cognitive). Our study focused on *how* and *why* families in Mafararikwa use social capital to navigate ANC, emphasizing the structural and cognitive dimensions of social capital.

Much of the current research around social capital and ANC access has been conducted in high-income countries.^5,10^ To our knowledge, no similar studies exist in (rural) Zimbabwe, although a few studies have examined social capital in non-ANC contexts.^7,23^ Elsewhere in SSA, research around this topic is limited. For instance, a fairly recent systematic review included only 13 studies—six of them from SSA and none from Zimbabwe.^10^ Moreover, current literature in SSA provides mixed findings, indicating that social capital is contextually bound.^24^ For instance, McTavish and Moore demonstrated in their study in rural Cameroon that increases in pregnant women’s social capital significantly increased the number of times the women contacted an ANC provider.^25^ However, the authors also found that neither social participation nor social network size was correlated with ANC utilization. Meanwhile, in their study in rural Ghana, Cofie *et al.* found that social network support affected women’s type of place of delivery, but the level of such support depended on whether the woman’s choice of place of delivery aligned with that of their social network.^26^ The paucity and mixture of evidence described above suggest a need for more context-specific studies to inform tailored interventions.

## Understanding Social Capital and Access to ANC in Rural Zimbabwe: Towards *Ubuntu* and ANC Pluralism Perspectives

Studies grounded in biomedical models generally define pregnancy as an individual *physical* condition and ANC as mainly physiological interventions.^1,12^ They use physiological indicators such as pregnancy-related illness (e.g., maternal malaria or anemia) and complications (preeclampsia, maternal deaths, etc.) to measure ANC and health outcomes.^1,12^ Within these conceptions, these studies typically define social capital as having the instrumental function to enhance the uptake or use of biomedical ANC.^25,26^ They consider favorably social capital and determinant factors that enhance the uptake or use of biomedical ANC while treating those that do not, or that promote the use of traditional or faith-based ANC as “harmful” or “barriers”.^27,28^ Yet in many parts of SSA, including rural Zimbabwe, people often utilize traditional, faith-based, and biomedical forms of ANC concurrently.^11^ Such ANC pluralism reflects holistic conceptions and multiple experiences of pregnancy, ANC, and health grounded, in large part, in *Ubuntu*.^13,15^

*Ubuntu* is a philosophy and way of life practiced among the Bantu people who originate from or live in SSA.^29,30^ Commonly expressed by the maxim *“I am, therefore, we are”*, *Ubuntu* describes a relationality whereby one’s existence is intricately intertwined with the existences of other beings in their community.^30,31^ In this context, community refers to harmonious relationships with other beings, where “other beings” encompass natural and spiritual entities, such as people, ancestral and other deceased spirits, and the physical environment (including land, plants, and animals).^31,32^ In terms of *Ubuntu*, health is multidimensional (physical, mental, psychological, emotional, social, environmental, spiritual), and being healthy entails maintaining a balance between one’s multiple health dimensions and relating harmoniously with other beings.^13,34^ Addressing any imbalance—such as illness or breakdown in social relationships—requires using multiple types of care, usually simultaneously.^11,13^ Such efforts involve the collaborative efforts of multiple actors (including care providers) within one’s family and local community.^13,29^

*Ubuntu* and ANC pluralism represent important frames for developing more inclusive and culturally appropriate understandings about social capital and ANC access in (rural) SSA. First, they infer that social capital facilitates access to multiple types of ANC, often concurrently, in various complex ways, consistent with a desire to achieve holistic health during pregnancy. Second, grounded in relationships, social capital and ANC are a way of life and have much more than instrumental value in many SSA cultures. Social capital is more than just a resource to be “used” to improve the uptake or use of ANC, while ANC is more than just a service “used” to achieve positive pregnancy and health outcomes. Last but not least, social capital and ANC are intricately intertwined and, in some cases, one and the same, e.g., African traditional or spiritual beliefs in the power of the ancestors to protect pregnant women from physical or spiritual harm can be defined as *both* cognitive social capital and forms of traditional or faith-based ANC.

Many studies grounded in a biomedical approach in SSA portray social capital as important in addressing barriers associated with the accessibility (e.g., long distances to health facilities), affordability (e.g., ANC or transportation costs), or the acceptability (religious beliefs, cultural practices, etc.) of health facility-based ANC.^10,25,26^ On the flip-side, the existing literature is ubiquitous with reports that traditional or faith-based forms of ANC are much more affordable and easily accessible since they are provided within families or local communities.^11,27,28^ Yet, a growing number of studies,^15,34^ including our previous work in Manicaland, Zimbabwe,^13^ indicate that families also face accessibility, affordability, and acceptability barriers in accessing traditional or faith-based ANC and receive support from their social networks and local community groups to navigate these challenges.

Ultimately, there is a need to go beyond biomedical conceptions and develop more inclusive, respectful, balanced, and fair understandings^35^ about social capital and access to ANC that reflect local socio-cultural realities in rural Zimbabwe and rural SSA. Employing *Ubuntu* and ANC pluralism can bring out these more nuanced, culturally grounded insights. The present study is an original contribution towards such analyses.

## Materials and Methods

### Design

We employed a case study design, which involves combining multiple methods to examine a phenomenon of interest in its natural setting, thereby generating an in-depth understanding of that issue, developing policy-relevant and actionable evidence, and charting further research pathways.^36^ We adopted an interpretive qualitative approach grounded in a social constructivist paradigm to explore contextually embedded meanings around social capital and ANC access.^37,38^ A case study approach allowed us to triangulate multiple data sources and capture the relational, social, and cultural dynamics underpinning social capital and ANC access. We employed several methods (more specific details provided below) to gain a comprehensive understanding of how families in Mafararikwa use social capital to access ANC. We were interested in what participants said and how their lived realities coalesced around *Ubuntu*, which aligns with a qualitative case study logic rooted in contextualism and meaning-making.^37,38^ Our findings will inform the creation of culturally-appropriate policies and further research in Mafararikwa and beyond.

### Setting

We conducted our study in Mafararikwa, rural ward 16 located in Manicaland, Zimbabwe (see Figure 1). In Zimbabwe’s government structure, a ward is the smallest administrative unit after districts, which are divisions of provinces. Manicaland is a predominantly rural province with a population of two million, located in Eastern Zimbabwe along the border with Mozambique.^18^ It has some of the largest volumes of refugees, ethnic and linguistic minorities, and populations that do not accept biomedicine, such as some apostolic sects.^23,34^ The sects just mentioned, particularly Johanne Marange—one of the largest apostolic movements in Zimbabwe—insist on using their own faith-based healing and spiritual interventions during pregnancy and childbirth over biomedical ANC.^34^ Their beliefs often shape community norms and practices around ANC, necessitating context-specific approaches to maternal health research.^13^

**Figure 1.**
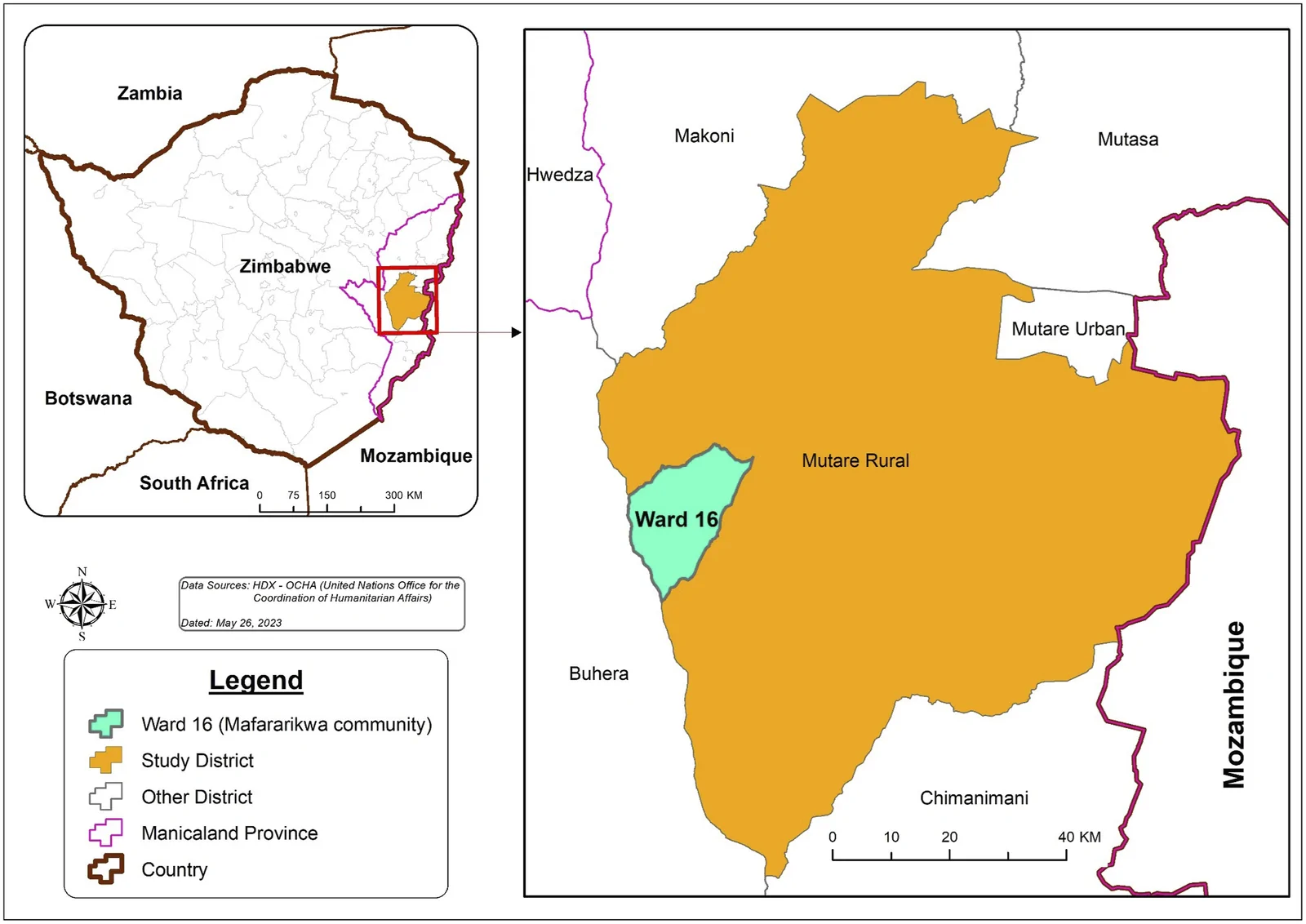
Map showing Mafararikwa in Manicaland, Zimbabwe.

Manicaland has a 71% absolute poverty rate, and most families there rely on subsistence farming as well as government and donor aid.^39^ Relative to Zimbabwe’s nine other provinces, it has lower maternal and child health outcomes, including the highest U5MR of 51 deaths per 1000 live births.^18^ Mafararikwa has a total population of 22,037 people.^18^ It has one public health facility, Zvipiripiri Rural Health Centre. It is linked to two referral hospitals: Sakubva District Hospital and Marange Mission Hospital. Sakubva District Hospital is located 120 km while Marange Mission is 40 km away from Zvipiripiri Rural Health Centre. Mafararikwa has a semi-arid climate that cannot sustain year-round maize (staple diet) production, leaving most of its families absolutely poor.^39^ It has some of the lowest maternal and child health outcomes compared to many other wards in Manicaland.^34,40^

### Data Generation

In Zimbabwe, national health policies are translated to sub-national policies at provincial and district levels and into health services at the ward level.^16^ We used a sampling frame aimed at informing ANC policies and practices at the district and ward levels. That frame was informed by a district-level health service providers register and key informant recommendations, guided by the aim to engage individuals who actively shape or experience ANC access in Mafararikwa. We framed our data generation methods within reciprocal research, a practical method for conducting ethical research with marginalized groups based on principles of human rights, empowerment, advocacy, and co-creation of authentic, actionable knowledge.^41^ This framing aligns with relational qualitative inquiry approaches rooted in *Ubuntu*.^35,42^ We used purposive sampling to identify participants who could speak to the diverse experiences of social capital use in ANC contexts, a technique commonly applied in reciprocal research.^41,43^ During the conceptualization of this study, we consulted nine senior health professionals (4 men, 5 women; 31–59 years) from the government and non-governmental organizations (NGOs) in Manicaland. These consultations, functioning as cognitive interviews,^44^ facilitated the identification of access to ANC as a research priority and the selection of the methods we later used to generate evidence addressing our study’s objectives.

We generated data for this study between April and June 2021 using four methods: in-depth interviews (IDIs), consultative sessions, focus group discussions (FGDs), and storyboarding (more details further below). Using purposive sampling,^43^ we selected a sample of 101 participants (51 women, 50 men) comprising key interest holders who design, provide, or receive ANC policies or services in Mafararikwa. We selected 101 participants based on their engagement in ANC as caregivers, health professionals, or community leaders. At the community level, selection was guided by an advisory committee and local community leaders to ensure a diverse sample. To protect participant anonymity given the small sample size, we only recorded age, sex/gender, and, for health professionals, sector. Given the cultural sensitivities surrounding pregnancy topics in Mafararikwa and Manicaland, we divided participants into men’s and women’s groups during data collection to foster greater openness and participation. Data generation with male participants was conducted by a male researcher (the first author) and a male research assistant (RA), while a female researcher and female RA conducted sessions with women participants. All four—researchers and RAs—are fluent in Shona (the main language in Mafararikwa/Manicaland) and English, and this proficiency was integral to data generation, transcription, and analysis.

IDI participants chose to use Shona and/or English, while all participants in consultative sessions, FGDs, and storyboarding opted to use Shona. We recorded all data generation sessions using digital audio recorders, complemented by detailed notes written down by RAs of key issues discussed and non-verbal cues of interest observed in each session. Discussion questions focused on how families leverage local informal support systems to access ANC in Mafararikwa. We asked similar questions across the IDIs, FGDs, and storyboarding, adapted slightly for depth and interactivity depending on the method and participants. See Appendix 1 for the IDI, FGD, and storyboarding topic guides. Combining IDIs, FGDs, and storyboarding enabled method triangulation, participant empowerment, and deeper engagement with nuanced, culturally complex issues, such as *Ubuntu* and ANC pluralism.^42^ This qualitative strategy aligns with best practices for exploring layered social phenomena.

### In-Depth Interviews

We conducted IDIs with 30 health or related professionals (13 men, 17 women; ages 31-63, mean age = 45 years) in Mafararikwa. 20 participants were from the public sector (i.e., government departments) and 10 from the private sector (i.e., NGOs). We selected these participants in consultation with Mutare’s District Medical Office using the district’s *Health Service Providers Register* as the sampling frame. The register contained details of health or related government ministries, local councils, and NGOs operating in Mafararikwa, including their scope of services, coverage areas, target groups, and contact details. A large majority of the organizations on this register provided biomedical healthcare services. From the register, we selected organizations that had been involved in developing or implementing ANC policies or services in Mutare for at least 6 months prior to this study. From those organizations, we selected the 30 IDI participants. The informed consent process included at least 2 weeks of reviewing a consent form detailing the study and participation parameters. Those who agreed to participate provided written consent by signing the consent form. The IDIs were conducted at the venue of each participant’s choosing (e.g., work office, quiet home space), with efforts made to ensure privacy and comfort.

We conducted and analyzed data from five IDIs first to develop interim themes, upon which we built 15 more IDIs. After conducting 20 IDIs, no new themes emerged; however, we conducted an additional 10 IDIs to further enrich our dataset. In international qualitative research standards, 25 to 30 IDIs can sufficiently saturate data.^45^ We used an interview guide containing open and closed-ended questions to facilitate focused conversations, probing, and follow-up while allowing participants to speak freely and openly.^46^ Open-ended questions enabled us to elicit detailed narratives while closed-ended questions enabled us to gather basic information for pattern recognition, consistent with qualitative interviewing best practices.^46^ All IDIs lasted between 20 and 80 minutes.

### Consultative Sessions

We conducted consultative sessions with 19 community-level key informants (9 men, 10 women; ages 20–67, mean age = 47 years) from Mafararikwa’s Health Centre Committee (HCC). These consultations functioned as cognitive interviews, enabling us to refine language, concepts, and thematic focus to ensure cultural resonance.^42,44^ In Zimbabwe’s Ministry of Health and Child Care (MoHCC) structure, HCCs comprise local public health professionals and representatives of local traditional and council leadership, traditional and faith-based care providers, schools, women, youth, and churches.^47^ Their main roles are to identify local priority health issues, raise their own resources to supplement those provided by the MoHCC, and address local health issues.^47^

We conducted our consultative sessions in two segments. In the first segment (1 hour), all participants and the research team discussed the study and planned for its implementation, including strategizing around participant recruitment and scheduling dates for data generation. The participants nominated an advisory committee of four members (2 women, 2 men) that the research team would work with during the fieldwork. Each committee member came from one of the ward’s four “regions” to ensure representation and timely information dissemination. In the second segment of the consultations, we split participants into men’s and women’s groups to brainstorm key themes to be discussed during later data generation activities. The men’s session lasted 45 minutes, and the women’s session lasted 52 minutes. The consultations helped focus and validate the study, interpreting abstract concepts into understandable, culturally appropriate terms.

### Focus Group Discussions

We conducted eight FGDs with 71 participants (37 men, 34 women; ages 19–68, mean age = 39 years) in Mafararikwa, divided as follows: (1) two FGDs with community-level key informants (10 men and 10 women each), (2) three FGDs with 8, 9, and 10 male parents or legal guardians (hereinafter, *caregivers*) apiece, and (3) three FGDs with 8 female caregivers, separately. The number of participants in each FG was sufficient to activate participation.^48^ Working with the selected study advisory committee and local village leadership, we recruited community-level key informants for our FGDs from the HCC in Mafararikwa. We selected caregivers to participate in FGDs from each of the 20 villages in Mafararikwa using a quota system. We invited each village head and their residents to identify two caregivers (1 woman, 1 man) to represent their village in the FGDs. We asked relatively larger villages to select a third participant, preferably a woman. Ultimately, 51 caregivers participated in the FGDs vs an initial target of 48 caregivers.

To participate, each FGD participant had to (1) have resided in Mafararikwa for at least 6 months, and (2) be expecting to be or have become a parent to a newborn in the 5 years before the scheduled date of their FGD. We used the 5-year reference period to capture a range of short and medium-term experiences while minimizing recall bias, consistent with international public health research.^3,18^ To further ensure fair representation, we asked participants to not be from the same family (e.g., spouses, close relatives) and encouraged that one of the two participants from each village be from a religious or other group that does not accept biomedical care. We also asked that selected participants be willing to participate and able to discuss issues related to local informal support systems and access to ANC in Mafararikwa. Our FGD sample comprised participants of diverse ages, sexes, villages, ethnicities, and religions, which we observed through some participants self-identifying themselves or from the religious or cultural attire they wore.

To collect data, we used a FGD guide containing both open and closed-ended questions to enable focused and probing discussions as well as open interaction among participants.^49^ We conducted and analyzed data from two FGDs (one with women, one with men) first to develop interim themes that we then took up in the remaining FGDs. No new themes emerged after six FGDs (three with women, three with men), but we conducted two more FGDs (one with men, one with women) to saturate our data.^48^ Our FGDs lasted between 77 and 133 minutes.

### Storyboarding

Storyboarding is a research method that emphasizes knowledge or evidence co-creation via consultation, relationship-building, and problem solving.^42^ We conducted storyboarding using the format developed by Pittaway and Bartolomei^41^ and implemented in over 20 countries worldwide with marginalized groups, including refugees. We conducted two storyboarding sessions, one with 10 men and another with 10 women (ages 20–67, mean age = 47 years). These were the same participants who participated in community-level key informant FGDs. We divided participants in each group into subgroups of two to three participants each and tasked them with preparing storyboards in response to questions pertaining to the study. Participants created the storyboards using verbal narration, drawings, and written contributions on flipcharts. Thereafter, we invited each sub-group to share their storyboard with the larger group, followed by a plenary.^41^

Storyboarding enabled participants and the research team to bring together key themes discussed previously in FGDs and to brainstorm strategies to strengthen local informal support systems and improve ANC access in Mafararikwa. The approach enabled visual and narrative expression, especially for participants who may not feel comfortable with formal interview structures or when discussing sensitive issues like pregnancy and care. The men’s storyboarding session was 55 minutes long, and the women’s 66 minutes long.

### Data Analysis

We transcribed all audio-recorded data into text and verified the texts by listening to their audio files while (re-)reading them. The two researchers (including the first author) and two RAs who collected data, as well as a contracted research consultancy organization, carried out the data transcription. In line with reciprocal research’s principle of accurate (re-)presentation of participants’ perspectives, we did not translate the transcripts from Shona to English to preserve meanings that could be lost in translation.^42^ This was particularly helpful because some Shona terms do not have direct English equivalents, such that, if translated, they can mean something different. For instance, there is no direct Shona equivalent for ANC. Participants often referred to ANC in mainstream health facilities as *kuskero*, cuing from the fact that expectant women are ‘scaled’ (weighed) as part of ANC in these facilities. Replacing *kuskero* with ANC could misinterpret participants’ understandings.

Following Braun and Clarke’s reflexive thematic analysis approach,^50^ we thematically coded and analyzed data using QDA Miner Lite software.^51^ Braun and Clarke’s approach allows for flexibility, depth, and critical reflexivity when analyzing rich, contextually complex data.^50^ It is ideal for studies like ours that aim to generate bottom-up themes while also reflecting on their theoretical framing—in this case, *Ubuntu* and ANC pluralism. Compared to other approaches such as grounded theory, which seeks theory generation, or phenomenology, which focuses on lived experience, thematic analysis enabled us to identify recurring patterns in the data while staying attuned to participant worldviews and cultural frames.

Reflexively and iteratively, we thoroughly familiarized ourselves with the data; generated initial codes; searched, reviewed, and identified themes; and finally developed the final write-up. Starting off inductively, we intuitively coded the data and identified common and divergent patterns and themes. Later, we deductively applied codes and themes from existing research, carefully noting any matches and tensions with codes and themes coming from our data. Finally, we combined our two rounds of analyses, carefully mapping out all overlaps and divergences as well as creating new codes and themes or new interpretations of existing codes and themes. With the first author coordinating, the other four co-authors (VS, JH, MS, MRC)—all fluent in English—engaged in this iterative analysis process, providing critical feedback that strengthened both the coding of data and development of thematic analysis. We applied thematic analysis across all datasets while also accounting for the unique dynamics of each method. Hence, consultations informed our analysis of key study concepts and language. We analyzed IDI data for individual narratives, FGD data for group perspectives, and storyboarding data for visual and collective representations of themes. We used one overarching codebook to ensure consistency, while allowing for method-specific sub-codes and themes.

### Enhancing Trustworthiness

To enhance the trustworthiness of our study and its findings, our RAs shared a summary of key issues discussed at the end of each consultative session, FGD, and storyboarding. After preliminary data analysis and interpretation, we met with a sub-sample of participants who had participated in data generation to discuss the initial analysis and findings.^52^ Throughout these activities, we invited participants to confirm, clarify, retract, or add any issues. During fieldwork, we also conducted routine debriefing meetings as a research team to reflect on emerging themes, redefine unclear concepts, identify areas to probe more, and address any logistical issues.^53^ We kept detailed notes/records of all decisions made and activities conducted, which enhanced consistency and transparency in our data generation and analysis.^52^ The four methods we used in this study (consultative sessions, IDIs, FGDs, storyboarding) represented forms of source, methodological, and data triangulation, enabling us to validate similar and conflicting themes.^54^ All authors of this article provided critical feedback throughout the study, facilitating investigator triangulation and reducing investigator or other biases.^54^

### Ethical Considerations

Ethical approvals were obtained from McGill Faculty of Arts Research Ethics Board (REB #20-11-005) and Medical Research Council of Zimbabwe (File #MRCZ/A/2708). The study received official written approvals from MoHCC in Zimbabwe at the national, provincial, and district levels, respectively. Mafararikwa’s HCC provided verbal approval, representing a form of collective consent consistent with *Ubuntu*. All participants participated voluntarily. Informed consent was ongoing, with participants able to validate or withdraw their participation or contributions at any point. We stored all data collected during this study securely and anonymously, with recordings encrypted and uploaded to a secure university cloud server, with all physical and digital data accessible only to the first author. In analyzing and reporting the data and findings, we excluded any identifying information mentioned during data collection.

## Results

We identified four main themes describing how families in Mafararikwa use social capital to access ANC: (1) Support through social networks and community groups, (2) Material and non-material support, (3) Reciprocal norms and sociocultural practices, and (4) Informal supports and care mixing dynamics. We present these themes below, along with purposely selected participant quotes to illustrate the themes (all indicated participant names are pseudonyms). The original, untranslated versions of the quotes are available from the authors upon reasonable request.

### Theme 1: Support through Social Networks and Community Groups

This theme describes how families in Mafararikwa draw support from their social networks, local groups, and community initiatives to engage with different types of ANC. Participants identified immediate family members (parents, spouses, older children) and extended family members (primarily in-laws, grandparents, aunts, uncles, siblings) as key sources of support for families navigating ANC access in Mafararikwa. They also identified friends, neighbours, health practitioners (traditional, faith-based, and biomedical), community resource persons (community health workers [CHWs], traditional leaders, etc.), and local community members as other key sources of support. These support mechanisms can be defined as *social networks*.

While the quote below illustrates how social networks support families to interact with mainstream health facility-based ANC, participants indicated similar perceptions about social network support within traditional and faith-based care systems:

[W]hen women are pregnant, carrying their unborn children, the relatives are also very helpful (…) If we look at the reasons why women may delay or not want to come to the hospital, some of them we said involve lack of resources, right? Relatives are the first people around (…) they help address those challenges. (Rembai, FGD, community-level key informant [CLKI], man).

Participants further shared that families in Mafararikwa draw support from local groups or community forums to access ANC. Such support mechanisms can be characterized as *civic participation forums*. Some key cited examples were church groups, mutual aid groups (e.g., internal savings and lending schemes [ISALS]), peer support groups (e.g., prevention of mother-to-child transmission of HIV [PMTCT] groups), and CHWs. Other participants specified community-level initiatives like community safety nets (e.g., *Zunde raMambo*) and community gardens as key sources of help for families navigating ANC access in Mafararikwa.

*Zunde raMambo* is a community-based safety net system in Zimbabwe traditional culture whereby community members pool resources – usually grain – to support food insecure families in times of need, such as droughts or economic hardships. It is practiced more in rural than urban areas. Some participants in this study clarified that not all villages in Mafararikwa have a *Zunde raMambo* and not all families participate in church groups, ISALS, PMTCT groups, or community gardens. However, all villages have at least one CHW who, although aligned to the biomedical healthcare system, supports all families regardless of their ANC preferences. This provides opportunities for ANC mixing, especially among those who do not accept or use biomedical ANC:

There are support groups that they [CHWs] do with expectant women and women who are breastfeeding, men whose spouses are expecting, men whose spouses are breastfeeding. Teaching them exactly what they can do from the moment they know they are expecting until the child is born, up to when the child grows to this or that age (…) It doesn’t matter if you go to the hospital or not, they help everyone. (Sipho, IDI, woman, public sector organization [PSO]).

Participants demonstrated that social networks, local groups, and community initiatives in Mafararikwa are intricately interconnected and analytically difficult to separate. For instance, some family members double as care providers, e.g., grandmothers or mothers-in-law who are traditional midwives. Likewise, members of community gardens or mutual aid groups are often friends, neighbours, or family members. These interactions result in families receiving multiple forms of support simultaneously when navigating ANC:

A woman may have relatives who do or believe in different things, all helping. Some may be some of those people who provide health education (…) When a woman is pregnant, they go and encourage (…) early maternity booking (…) Others, like elderly women, know about traditional herbs, they recommend to the woman. “Why don’t you try this? We used it in our time.” (Jabu, IDI, man, NGO).

### Theme 2: Material and Non-material Support

This theme describes the material and non-material supports that families in Mafararikwa draw from their social networks, local groups, and community initiatives to interact with different forms of ANC. Participants opined that families leverage ***material support,*** including financial assistance, from their social networks, local groups, and community initiatives to access ANC in Mafararikwa. Such financial support included money for expenses like maternity registration fees, ANC contact or delivery care charges, outsourced services (e.g., ultrasound scans), medication, transportation, food, and clothing, among others. While the following excerpt illustrates these findings within traditional and faith-based care systems, some participants understood that families leveraged financial support from their social networks, local groups, and community initiatives to access ANC in other healthcare systems in Mafararikwa:

When I was pregnant with my child who is here, the child was breached. The midwives [at chitsidzo] tried their best to turn the child but in vain, until they referred me to another midwife in Buhera. That midwife helped me. But to go there, I needed money for transport and food. My husband supported me. (Netsai, FGD, woman caregiver).

Families in Mafararikwa leverage material support, such as *specific goods* like food, clothing, or transportation, from their social networks, local groups, or community initiatives to access ANC. For instance, some participants explained that some families cannot afford to send their expectant women to waiting mothers’ shelters (places at public health or traditional or faith-based care facilities where expectant women who live far away can stay as they await giving birth) due to household food limitations. In such situations, families must divide their food into two portions: one for the family members remaining behind and the other for the expectant women going to the waiting mothers’ shelter. Social networks help with food items in this regard.

The excerpt below cites another example of goods given to families via social networks, local groups, or community initiatives to access ANC in Mafararikwa. It is in a biomedical care setting, but similar perceptions were reported in faith-based or traditional care systems too:

[S]ome of our hospitals are far away (…) If you get to a point where you are having labour pains, they [family or community members] can help you with transportation, like an ox-drawn cart, to ferry you to the hospital (…) Even her baby’s old clothes, from when her baby was young, my friend can give me as well. (Thembiso, FGD, woman caregiver).

Families in Mafararikwa leverage ***non-material support*** in terms of *social support* from their social networks, local groups, or community initiatives to interact with ANC. Some key examples of social support include help with domestic work (e.g., fetching water, cleaning the house), household economic work (e.g., tilling or harvesting the fields), and childcare (e.g., minding one’s children). Participants stated that most people in Mafararikwa perceive women as responsible for domestic, household economic, and childcare work. Hence, balancing such duties and pregnancy, childbirth, and ANC access can be difficult for women.

Social support, like the kinds mentioned above, can free up time for expectant women to, for instance, attend their ANC appointments or rest. Participants understood that these situations apply to expectant women regardless of their preferred type(s) of ANC:

When it comes to [domestic] work, as you get to nine months, maybe your back hurts, (…) churchmates or neighbours can come to help, say in this harvesting time (…) [L]et us help you harvest this, let us put this stuff here, put this stuff there (…) You also get time to rest, or go to the hospital or chitsidzo, if that is where you go, because the workload has lessened. (Sheba, storyboarding CLKI, woman).

*Moral support* is another form of non-material support that families draw on from their social networks, local groups, or community initiatives to access ANC in Mafararikwa. Some key examples of moral support participants cited are escorting expectant women for their ANC contacts or birthing, visiting expectant women when they are not feeling well or to spend time with them, and providing counselling during stressful situations. Participants perceived that moral support helps expectant women and their families feel that “*they are not alone*”, which also enhances their mental and emotional well-being during pregnancy. The heterogeneity of social networks or local groups to which families or expectant women in Mafararikwa are connected means that they often receive different types of moral support:

The father, since it’s his family, will often accompany their partner for their check-up, whether here [public health facility] or at the traditional midwife’s. Maybe sometimes he is not there, the woman is accompanied by relatives, be it an aunt, or someone else, or an uncle (…) [Y]ou may get a call to say your wife has taken to the hospital (…) [I]t is those relatives who would have taken her there. (Thabo, FGD, man caregiver).

Families in Mafararikwa leverage *information* as a form of non-material support from their social networks, local groups, or community initiatives to engage with ANC. Participants conveyed that such information may include where to access specific services or providers, what to eat to stay healthy in pregnancy, or where to give birth. They noted that information support often comes from different sources within families’ or expectant women’s networks or groups, which facilitates concurrent use of different types of ANC:

In our traditional culture, you may meet a grandmother (…) who can say, “You! I see when you’re walking, or your stomach is a bit strange, do you ever go to a [traditional] midwife to get checked? (…) Oh, you, your stomach is not well. Go and get checked by midwife so and so.” (…) It does not mean you stop going to the clinic, but it is also best to heed the grandmother’s recommendation. (Nyasha, FGD, woman caregiver).

*Spiritual support* is another form of non-material support participants reported that families leverage through their social networks, local groups, or community initiatives to access ANC in Mafararikwa. Participants perceived that families in this ward consider spiritual support as an important dimension of care during pregnancy or childbirth, regardless of one’s preferred type of ANC. Some families or expectant women access special protection rituals, prayers, consecrated ornaments, or traditional medicines to cure illnesses or enhance mother and child health. Some church members “fellowship” (e.g., through home visits) with their expectant colleagues to demonstrate their solidarity and spiritual support. Some traditional or spiritual care providers, religious leaders, or senior family members provide counseling or emotional support to families to navigate stressful situations during pregnancy or childbirth:

[E]ven church folks, if you invite them, those nearby will visit you. Others say prayers for you (…) Women’s fellowship groups from your church too (…) [E]ven Satan or evil spirits run away, you stop worrying about whether you and your child will survive this (…) Churches forbid traditional spiritual practices, but people still do it because some things require traditional healers. (Namatai, FGD, woman caregiver).

Some families leverage *ANC services* as a form of non-material support from their social networks who are healthcare practitioners in Mafararikwa. These situations demonstrate blurred professional-personal boundaries. For instance, some [grand]mothers or mothers-in-law are also traditional or apostolic sect midwives or spiritual or traditional faith healers who provide ANC within their practice expertise to their expectant [grand]daughters, daughters-in-law, sisters, etc. Likewise, some public health practitioners have families, friends, neighbours, or churchmates, etc., who may call on their support for ANC. Care providers act as information and referral hubs to other needed care, which often transcends multiple ANC systems:

The community health workers help them [expectant women] (…) to register early. They follow up, “Are you going for your ANC contacts? Have you been immunized?” (…) Some of the mothers are their relatives, they are trusted in their villages (…) They don’t refer mothers to local [traditional] midwives. I think they refer spiritual matters because they know their villages well, but that is not as part of our [ANC] protocols. (Mutsa, IDI, woman, PSO).

### Theme 3: Reciprocal Norms and Sociocultural Practices

This theme depicts how families leverage reciprocal actions, norms of respect and courtesy towards expectant women, mutual trust, and socio-cultural practices to engage with diverse ANC types in Mafararikwa. Because of their social ties, families in Mafararikwa feel emotionally and socially invested in helping others. They also trust that others will help them when they need support, including when navigating access to ANC. Such reciprocal actions, which reflect *social cohesion*, demonstrate African communitarian relationships grounded in a shared sense of identity and acts of solidarity. Participants demonstrated such understandings through responses like “*this is what we do*”, “*this is who we are*”, “*this is our thing*”, and “*this is for us*”. They did not seem to have different perceptions based on a specific type of ANC system. On how and why families help each other to access ANC in Mafararikwa, one participant reasoned as follows:

[T]his issue we are talking about, it creates team spirit, team working (…) [W]e develop a we-feeling. This is our thing. When that happens, love develops (..) [Y]ou will see that even these [pregnancy-related] deaths reduce because we are working together, all of us as a community. (Tembo, FGD, CLKI, man).

Participants also mentioned that norms of respect, courtesy, and preferential treatment of expectant women ensure that people in Mafararikwa support expectant women whenever they can for positive pregnancy outcomes. For instance, expectant women usually get served first during community or social activities (e.g., food aid distribution programs). Childbearing and motherhood, mainly in marriage contexts, are revered in Mafararikwa and often translate into helping practices towards expectant women. This study revealed that the norms and behaviours presented above, which also reflect social cohesion, did not vary by ANC system:

[W]hen a person is pregnant, they [family or local community members] give you that emotional support, indicating their joy at the prospect of having (…) a new family member. Traditionally, the childbearing role of a woman is highly appreciated. So, every now and then, if it is even possible, she may be spoiled (…) Some support so that everything goes smoothly, and others can be referral points, be it [to] traditional or modern [care]. (Raison, IDIs, man, PSO).

Participants expressed that families in Mafararikwa prefer to receive or provide support to access ANC from/to people with whom they have a mutual, trust-filled relationship. Mutual trust, which enhances social cohesion, was mentioned as especially important to families during the early months of pregnancy as they prefer to leverage help from close, well-trusted networks to protect expectant women and unborn children from spiritual harm:

You don't tell anyone [about your pregnancy] just like that. You tell just a few people you trust, whom you know are truly there to help you (…) [A]lso, go to a [traditional] midwife whom you know can keep your confidence, she helps you with herbs to caste evil spirits or strengthen your child. (Mandi, FGD, woman caregiver).

The present study revealed that socio-cultural factors are another form of social capital families leverage to access ANC in Mafararikwa. Such factors demonstrate and build social cohesion. For example, some members of some apostolic sects felt that, due to their religious beliefs, they help each other to use faith healing methods prescribed in their sects during pregnancy or childbirth. In some instances, this entails traveling to consult with spiritual care providers or midwives within their apostolic systems but outside Mafararikwa. However, some participants noted that apostolic sect beliefs play a complex role in shaping ANC access in Mafararikwa. Some beliefs, according to these participants, restrict engagement with biomedical ANC, while others adopt a pluralistic approach by drawing on different ANC types.

Other participants identified traditional cultural practices that bring families together to support expectant women. One often cited practice is *masungiro*, whereby a married woman expecting their first child goes back to their maiden family during the last trimester of pregnancy until about a month after giving birth. During that time, the woman receives guidance and support to navigate pregnancy, childbearing, and access to care, be it faith-based, traditional, or biomedical care, from her [grand]mother or other senior women within the confines of her maiden family. Some participants added that some families continue such practices in later pregnancies, mainly when the family or expectant woman is still relatively younger:

Like us young fathers, when my wife is pregnant for the first time or the second time, or something. When her [delivery] days are approaching, (...) I can take her to her parents and ask them to stay with her and help her until she gives birth to our child (…) I can make sure that everything she needs to go the clinic is in place, but I may not know well how they care for an expectant woman in their own culture (…) [T]hat is where her family comes in. (Pepukai, storyboarding, CLKI, man).

Some families, local groups, or communities encourage their members to perform socio-cultural practices that ensure positive pregnancy and childbirth experiences, including access to care. Such encouragements demonstrate *informal social control* as a form of social capital. The apostolic religious beliefs, spiritual healing methods, and *masungiro* presented above are some key examples of socio-cultural practices that are encouraged in Mafararikwa.

Another key practice severally cited is home births. Encouraged in some families as part of their cultures, home births enable [grand]mothers, mothers-in-law, or other senior women to support expectant women in navigating pregnancy, childbirth, and access to ANC, which may or may not include different types of care. They enable families to perform their family rituals (e.g., disposing the placenta or umbilical cord, welcoming the new-born into the family) which may be difficult to perform when one delivers in a mainstream health facility.

Participants’ responses revealed that while some families, local groups, or communities encourage socio-cultural practices related to ANC among their members, families leverage those practices to access ANC from multiple care systems in Mafararikwa. Families, local groups and community members have different ANC needs and preferences, and, as such, support others to interact with multiple types of ANC, often simultaneously. While the following excerpt provides additional context to these findings using the example of traditional herbal use in pregnancy, we observed similar perceptions in many other practices, including in the apostolic sects, *masungiro*, and home birth situations presented above:

In the villages where we live in this community, as expectant women, we are taught by grandmothers in the community that, in terms of our culture, we must prepare the child’s path when you are ready to give birth. We get told to use such and such [herbs] to prepare that passageway (...) They will say go to the hospital if you want but make sure to use this too. Our own mothers gave birth to us using these same practices, so they work. (Kundai, FGD, woman caregiver).

While they are presented here somewhat distinctly, participants in this study described the above-outlined reciprocal actions, norms of respect and courtesy to expectant women, mutual trust, and local socio-cultural practices in Mafararikwa in nuanced and integrated ways. Hence, reciprocal actions were perceived as shaping and being shaped by mutual trust, just as socio-cultural practices were seen as deeply interwoven with respect and courtesy norms. Furthermore, these mechanisms were expressed as intertwined with the mediums that convey and reflect them – social networks, local groups, or community initiatives. Community gardens, for example, demonstrate and enact unity and helping actions in ANC access settings:

That’s where our humanness lies (…) that we cannot let anyone struggle because they are pregnant. Most of the time, people who experience challenges with their pregnancies, usually the husband is not there or there are no resources to sustain themselves. So, as a community, to show that we are united, to be like family, we help each other. (Khumbulani, storyboarding, CLKI, man).

### Theme 4: Informal Supports and Care Mixing Dynamics

This theme describes how local informal supports enhance or limit families’ interactions with different types of ANC in Mafararikwa. Participants’ responses demonstrated that forms of social capital that are based on a biomedical approach support families to access biomedical services more than other types of ANC in Mafararikwa. Conversely, social capital mechanisms grounded in African indigenous worldviews help families engage with traditional or faith-based ANC more than biomedical ANC. Yet, there is also the “messy middle”, where families or expectant women access multiple types of ANC largely due to leveraging help from social capital mechanisms aligned with different ANC systems. Participants perceived that most families or expectant women in Mafararikwa are in this “messy middle”:

I’d say where most mothers stand, we try all sorts of things. At the hospital– (Others interject: I’m there!). At the traditional– (Others interject: I’m there!). At the church as well, we are– (Others interject: We are there!) (…) In all those places, I have friends or relatives, or colleagues from the church, who help me. If I want this thing, this person helps me get it. If I want that thing, I'll go to that person. You see? (Faith, FGD, CLKI, woman).

Some participants added that some social networks or groups restrict some populations from accessing (their preferred) ANC in Mafararikwa. Such networks or groups use coercive informal social control mechanisms to encourage their members to conform with ANC practices usually observed in those networks or groups. A frequently cited example entails some apostolic sects that use threats or acts of ostracism, divorce, violence, ridicule, or shaming to ensure their members use only faith healing methods recommended in these sects (this does not necessarily mean that members of these apostolic sects have no option but to comply with their sects’ requirements. In fact, participants in this study opined that some members of these sects openly or covertly access other forms of care, thereby defying their sects’ rules. The authors of this article have left a detailed analysis of this complex issue to a future paper).

Another example of coercive informal social control highlighted in this study entails the perceived growing “pressure” to access ANC and deliver in public health facilities, alongside the shaming of non-biomedical forms of care and home births in Mafararikwa:

My husband and I wanted to give birth at home. My mother-in-law (…) had all her children at home. That is what they do in their family (…) But, due to the fact that everywhere it is being encouraged to give birth at the hospital, I gave birth there (…) Nowadays if you give birth at home, people will see you as crazy, you will feel ashamed in public [laughing]. (Pipilota, FGD, woman).

## Discussion

Guided by *Ubuntu* and ANC pluralism perspectives, we examined how families use social capital to access ANC in Mafararikwa. Our findings indicate that, to access ANC, families in Mafararikwa draw material (money and goods) and non-material supports (social, moral, information, and spiritual support and ANC services) from social networks, local groups, and community initiatives, and leverage reciprocal actions, norms of respect and courtesy towards pregnant women, mutual trust, and socio-cultural practices. These local informal supports enable families to engage, often simultaneously, with traditional, faith-based, or biomedical ANC. Our findings reflect relationships and ANC pluralism grounded in *Ubuntu* that point to conceptions of and a desire for more holistic health and ANC in rural SSA. We discuss these findings below, underlining how they relate and contribute to existing knowledge.

The finding that families in Mafararikwa draw support from their social networks, local groups, and community initiatives to access biomedical ANC aligns with a few previous studies in rural Zimbabwe, although they focused on non-ANC topics.^7,23^ This finding is also consistent with existing research focusing on health facility-based ANC in some rural areas in SSA, such as in Cameroon^25^ and Ghana.^26^ As well, the finding that families in Mafararikwa draw on support from the above-mentioned local informal systems to access traditional or faith-based ANC builds on previous research in Mafararikwa^34^ and elsewhere in rural Zimbabwe^55^ and rural SSA.^2,25,26^ Yet, many of these current studies do not define the support mechanisms we analyze as social capital. They often construe support for traditional or faith-based ANC negatively, treating them as barriers rather than enablers of holistic care. Our findings complicate this picture, demonstrating how support for all forms of care can simultaneously represent cognitive social capital.

The finding that families in Mafararikwa leverage their reciprocal actions, mutual trust, norms of respect and courtesy towards pregnant women, and socio-cultural practices to access biomedical ANC aligns with previous literature in Mafararikwa^7,34^ and elsewhere in rural Zimbabwe^23,55^ and rural SSA.^28,56^ Yet, unlike the present study, these current studies often define the supports under present analysis as “harmful” or “barriers” for promoting engagement with traditional or faith-based ANC. This is because they typically privilege biomedical ANC in relation to African traditional and faith-based forms of ANC. The few that recognize or embrace local informal supports that promote interaction with traditional or faith-based care practices also do not typically define these supports as social capital.^2,55^ Our findings suggest that the reciprocal actions, mutual trust, respect and courtesy norms towards pregnant women, and socio-cultural practices that enable families in Mafararikwa to engage with non-biomedical ANC can be described as cognitive social capital. Indeed, they are local strengths, not mere norms, beliefs, or practices.

Our findings collectively highlight the layered and relational nature of social capital in Mafararikwa. We draw attention to our inference that, when we employ *Ubuntu* and ANC pluralism perspectives, we develop new meanings around social capital and ANC access in Mafararikwa. The different forms of social capital that families in this community draw support from to access multiple forms of ANC reflect relationships (defined as identifying with and acting in solidarity with others^31^) as well as multiple experiences and holistic conceptions of pregnancy, ANC, and health.^13,15^ In Mafararikwa, at least, social capital transcends multiple systems of health and ANC, facilitating holistic health outcomes. Families experience pregnancy, ANC, and health in contexts of community, coordinate and cooperate with each other, share a sense of togetherness, and exhibit sympathetic altruism towards one another. Again, a core rationale for all this reverts to, as one participant put it, *“this is where our humanness lies”*. These findings contrast with many current studies in Mafararikwa and across rural SSA that focus on social capital within biomedical care settings and emphasize the achievement of physical health outcomes.^10,11^

Our findings suggest complexities around differentiating between various types of social capital. The current literature generally defines structural and cognitive social capital as distinct concepts.^5,21^ However, our findings indicate, for instance, that families in Mafararikwa have members of their social networks who also participate in the local groups or community initiatives that support them. These same members also engage with the families reciprocally, share trust, act respectfully and courteously towards them during pregnancy, or share socio-cultural practices related to ANC. Furthermore, we observed that the lines between some forms of social capital and ANC were obscured in Mafararikwa. For example, some grandmothers or mothers-in-law (social networks) were portrayed as traditional midwives (both social networks and ANC providers) as well. Our prior work in Mafararikwa^13^ and other similar studies in rural Zimbabwe^7,55^ as well as rural SSA,^2,26^ indicate that families in SSA contexts consider the trust or relationships they share with their traditional midwives as a *form* of ANC. Ultimately, our findings suggest more holistic and relational understandings of social capital and ANC in Mafararikwa, and by implication, across rural SSA, that do not quite align with conventional reductionist definitions.

Findings in our study suggest that local informal supports enable families in Mafararikwa to navigate challenges that would otherwise prevent them from accessing their preferred types of ANC. The findings align with previous research in Mafararikwa^7,34^ and other rural areas in Zimbabwe,^23,55^ as well as in SSA.^25,26^ However, much of this research focuses on mainstream health facilities, giving the impression that social capital is a relevant concept only insofar as it applies to access to biomedical ANC. Meanwhile, our study indicates that, in pluralistic ANC contexts like Mafararikwa, families also experience challenges in accessing traditional or faith-based ANC and draw support from their social capital to navigate these challenges. This situation reflects multiple experiences and holistic conceptions of social capital, pregnancy, ANC, and health grounded in *Ubuntu*. Hence, when we consider *Ubuntu* and ANC pluralism, we observe that mechanisms that help families in Mafararikwa to engage with traditional or faith-based ANC can be defined as forms of social capital.

The finding that some local informal supports in Mafararikwa prevent pregnant women or families who want to use health facility-based ANC from accessing such care resonates with previous studies in Mafararikwa^7,34^ and other rural settings in Zimbabwe^23,57^ and SSA.^27,28^ We observed these findings in terms of, for example, informal social control among apostolic sects that do not accept biomedical care and families who maintain or insist on home birthing traditions. Existing research grounded in biomedical approaches often define these mechanisms as “harmful” or “barriers”.^27^^.^^28^ Further, the finding that some aspects of social capital impede families or women from utilizing preferred forms of traditional or faith-based ANC in Mafararikwa aligns with a thin but growing body of literature in Mafararikwa^34^ and elsewhere in rural Zimbabwe^58^ and rural SSA.^15^^,^^56^ Like ours does, these studies report, for instance, that some social networks or norms dissuade the use of or look down on families or women who use traditional or faith-based ANC. Our study thus highlights the complex ways in which social capital may restrict access to ANC in pluralistic care contexts like Mafararikwa. Some scholars have referred to such aspects as the “dark side” of social capital,^59^ although we will refer to them as the negative impacts.

### Policy, Practice, and Research Implications

Efforts to strengthen local social capital to enhance access to ANC must start from where families are at in Mafararikwa. This means recognizing and embracing relational and communal support networks and systems that shape and are shaped by families’ multiple experiences and holistic conceptions of pregnancy, ANC, and health grounded in *Ubuntu*. This is not the same as condoning social capital mechanisms that restrict individuals, families, or local communities from accessing their preferred forms of ANC. Rather, all interest holders—including families, pregnant women, and care providers—should engage in genuine dialogue, learning, and information-sharing based on mutual respect, empathy, solidarity, and harmony.^15,35^

ANC policies and practices in Mafararikwa and, by extension, rural Zimbabwe and rural SSA must shift from the dominant biomedical model that treats anything that does not promote the uptake or utilization of biomedical ANC as “harmful” or “barrier”. They must employ a collaborative co-existence approach that embraces complexity, multiple realities, holistic health and healthcare, and complementarity.^13^ This approach can prevent a priori judgements about what the best ANC is, ensure that families’ and individual pregnant women’s ANC preferences are considered, and leverage local strengths to enhance access to different forms of ANC.^13,15^ To facilitate this, *Ubuntu* can be used as an ideal core framework.^24,31^

Efforts to strengthen local informal support systems and enhance access to ANC in Mafararikwa, rural Zimbabwe, or rural SSA must consider the complex interconnections between different forms of social capital. They must consider the full extent of the networks and relationships in which families or expectant women who need support to access ANC live. Often, current interventions are segmented, such as support groups for expectant women that lack efforts to engage other interest holders within the women’s networks (spouses, grandmothers, mothers-in-law, etc.) who play critical roles in supporting the women’s engagement with ANC.^2,56^ Supporting expectant women in navigating access to ANC without factoring in their broader networks or relationships can fail to achieve the desired outcomes.

Future research should build on this study by conducting longitudinal analyses of how social capital mechanisms evolve over time in relation to ANC preferences in Mafararikwa and other similar settings across SSA. More comparative, qualitative, and quantitative studies across wards or regions are needed to deepen the understanding of how access to ANC varies by faith or ethnic group. Additionally, we recommend mixed-methods research to explore the influence of different social capital forms on the use of various types of ANC, informing policy interventions that are both context-specific, culturally resonant, and scalable.

## Limitations

In this study, we employed an *Ubuntu* approach, which is generally applicable to SSA, and some of our findings align with current research in this region. However, we cannot claim full generalizability of our findings to other areas in Mafararikwa, rural Zimbabwe, and rural SSA due to our use of a qualitative approach and case study design as well as the context-boundedness of social capital, unless those other areas are sufficiently similar to our research community.^38^ Using a robust sampling strategy, we selected diverse participant groups who contributed richly to this study. Yet, given our small sample size, we may have missed other important perspectives due to the potential exclusion of other participants.

Although we did not receive any complaints about our participant recruitment strategy during or after the study, we acknowledge that our study may have retained some bias. For example, based on their extensive experience of working or conducting similar research in Manicaland, members of our research team shared that there is a possibility, albeit slim, that some local village heads might exercise discretion by nominating specific individuals whom they believe would best represent their communities in the research project. If this happened, especially given the small village-level participant quotas we implemented, some capable or willing participants may have been excluded. Finally, our study captured recent understandings only given its cross-sectional design. It does not reflect longitudinal understandings per se, though some older participants shared useful anecdotes about temporal changes in social capital and access to ANC in Mafararikwa that enriched our study.

## Conclusion

In this study, we examined how families leverage social capital to access ANC in Mafararikwa. We provided a comprehensive understanding of the various complex ways in which social capital enables (or restricts) families in Mafararikwa to access traditional, faith-based, or biomedical ANC, thereby making an effective and original contribution to existing knowledge. By considering *Ubuntu* and ANC pluralism, we highlighted the relationships and holistic conceptions surrounding health and healthcare that shape how families support each other to access their preferred types of care in Mafararikwa. Policies and practices aimed at strengthening local informal support systems and enhancing access to ANC must start from where families are and employ a collaborative coexistence approach that centres *Ubuntu*, ANC pluralism, and multiple realities in Mafararikwa and beyond.

## Supplemental Material

Supplemental Material - That’s Where Our Humanness Lies”: How Families Leverage Social Capital to Access Antenatal Care in Rural, Eastern Zimbabwe Supplemental Material for That’s Where Our Humanness Lies”: How Families Leverage Social Capital to Access Antenatal Care in Rural, Eastern Zimbabwe by Anthony Shuko Musiwa, Vandna Sinha, Jill Hanley, Morten Skovdal, Mónica Ruiz-Casares in Community Health Equity Research & Policy

## Data Availability

The data generated and analyzed in this study are not publicly available to uphold the privacy and confidentiality of participants, as agreed to by participants as part of their informed consent. The data are available from the corresponding author on reasonable request.*
