# Supplementary material for: That’s Where Our Humanness Lies”: How Families Leverage Social Capital to Access Antenatal Care in Rural, Eastern Zimbabwe
Source: Community Health Equity Res Policy. 2025 Nov 20;47(2):197–214. doi: 10.1177/2752535X251395358 (PMC13752226; doi:10.1177/2752535X251395358)
Supplement: Supplementary file 1 [file sj-pdf-1-qch-10.1177_2752535X251395358.pdf]

Appendix 1. Topic guides used in the study on social capital and access to antenatal care in Mafararikwa, rural Eastern Zimbabwe.

Addendum 1.1. In-depth interview guide

Expected participants: District-level healthcare policymakers, managers, senior practitioners

Interview questions

Let us start by discussing the nature of access among pregnant women and their families to programs and support that maintain their health and wellbeing and that of their unborn children provided in or through public health facilities in Mutare rural district.

1. When a pregnant woman goes to a clinic or hospital in this district, what programs or support does she ordinarily receive there to maintain her health and wellbeing and that of her unborn child?
2. In your opinion, the things you mentioned that a pregnant woman ordinarily receives when they go to the clinic or hospital in this district:
   a) Are they available in these clinics or hospitals as they should be?
   b) How do pregnant women and their families navigate the clinic or hospital once they get there to access these things?
   c) Can pregnant women and their families afford to pay for these things?
   d) Are these things meeting the needs of pregnant women and their families?
   e) Are pregnant women and their families satisfied with these things?
3. What barriers, and the determinants of these barriers, do pregnant women and their families experience when accessing programs or support from public health facilities in this district to maintain their health and well-being and that of their unborn children?

Now, let us discuss the nature of access among pregnant women and their families to alternative forms of care that maintain their health and wellbeing and that of their unborn children provided outside clinics and hospitals in Mutare rural district.

4. Apart from going to the clinic or hospital, are there any other healthcare strategies that pregnant women and their families in this district use to maintain their health and wellbeing and that of their unborn children?
5. Why do some pregnant women and their families in this district choose other forms of healthcare, besides going to the clinic or hospital, to maintain their health and wellbeing and that of their unborn children?
6. In an effort to protect their health and well-being and that of their unborn children, do you think pregnant women and their families in this district both go to the clinic or hospital and use other alternative forms of care, or do they prefer one form of care to another?
7. What barriers, and the determinants of these barriers, do pregnant women and their families experience when accessing alternative forms of care that maintain their health and well-being and that of their unborn children in this district?

Let us discuss the various strategies that pregnant women and their families in Mutare rural district use to address the barriers they experience when accessing programs and support that maintain their health and well-being and that of their unborn children.

8. How are relatives and friends helping pregnant women and their families in this district to access programs or support that maintain their health and wellbeing and that of their unborn children?
9. How are local communities helping pregnant women and their families in this district to access programs or support that maintain their health and wellbeing and that of their unborn children?
10. What cultural norms and practices enable people in this district to unite and help pregnant women and their families to access programs or support that maintain their health and wellbeing and that of their unborn children?
11. How are government and NGOs helping pregnant women and their families in this district to access programs or support that maintain their health and wellbeing and that of their unborn children?
12. Apart from relatives, friends, neighbours, local communities, government and NGOs, are there any other ways in which pregnant women and their families in this district receive help to access programs or support that maintain their health and wellbeing and that of their unborn children?

Now, I would like to understand your perspective of what programs and support that maintain the health and well-being of pregnant women and their unborn children in Mutare rural district should be or look like.

13. Imagine a woman in this district has just learned that she is pregnant, what do you think she should do or receive to protect her health and wellbeing and that of her unborn child?
14. Why is it important for pregnant women and their families in this district to have access to those programs or support that maintain their health and wellbeing and that of their unborn children that you mentioned?

Appendix 1.2. Focus group discussions guide

Expected participants: Members of the ward Health Centre Committee and adult caregivers (parents and/or guardians ages 18 years or older)

Discussion questions

Let us start by discussing the nature of access among pregnant women and their families to programs and support that maintain their health and wellbeing and that of their unborn children provided in or through public health facilities in this ward.

1. When a pregnant woman in your family or ward goes to the clinic or hospital, what programs or support do they ordinarily receive there to maintain her health and wellbeing and that of her unborn child?
2. The things you said that a pregnant woman in your family or ward should receive when she goes to the clinic or hospital, are they available at the clinics or hospitals in this ward or in Mutare rural district?
3. What barriers, and the determinants of these barriers, do pregnant women and their families experience when accessing programs or support from public health facilities in this ward and in this district to maintain their health and well-being and that of their unborn children?

Now, let us discuss the nature of access among pregnant women and their families to alternative forms of care that maintain their health and wellbeing and that of their unborn children provided outside clinics and hospitals in this ward and Mutare rural district.

4. Apart from going to the clinic or hospital, are there any other healthcare strategies that pregnant women and their families in this ward use, to maintain their health and wellbeing and that of their unborn children?
5. Why do some pregnant women and their families in this ward use other forms of healthcare, besides going to the clinic or hospital, to maintain their health and wellbeing and that of their unborn children?
6. In an effort to protect their health and well-being and that of their unborn children, do you think pregnant women and their families in this ward both go to the clinic or hospital and use other alternative forms of care, or do they prefer one form of care to another?
7. What barriers, and the determinants of these barriers, do pregnant women and their families experience when accessing alternative forms of care that maintain their health and well-being and that of their unborn children in this ward?

Let us discuss the various strategies that pregnant women and their families in this ward use to address the barriers they experience when accessing programs and support that maintain their health and well-being and that of their unborn children.

8. How are relatives and friends helping pregnant women and their families in this ward to access programs or support that maintain their health and wellbeing and that of their unborn children?
9. How are local communities helping pregnant women and their families in this ward to access programs or support that maintain their health and wellbeing and that of their unborn children?
10. What cultural norms and practices enable people in this ward to unite and help pregnant women and their families to access programs or support that maintain their health and wellbeing and that of their unborn children?
11. How are government and NGOs helping pregnant women and their families in this ward to access programs or support that maintain their health and wellbeing and that of their unborn children?
12. Apart from relatives, friends, neighbours, local communities, government and NGOs, are there any other ways in which pregnant women and their families in this ward receive help to access programs or support that maintain their health and wellbeing and that of their unborn children?

Now, I would like to understand your perspectives of what programs and support that maintain the health and well-being of pregnant women and their unborn children in your ward and in Mutare rural district should be or look like.

13. Imagine a woman in your family or ward has just learned that she is pregnant, what do you think she should do to maintain her health and wellbeing and that of her unborn child?
14. Why is it important for pregnant women and their families in this ward to have access to programs and support that maintain their health and wellbeing and that of their unborn children?

Appendix 1.3. Storyboarding guide

Expected participants: Members of the ward Health Centre Committee (HCC)

Storyboarding questions

1. What barriers do pregnant women and their families in this ward experience when accessing programs or support that maintain their health and wellbeing and that of their unborn children?
2. What are the determinants of the barriers that pregnant women and their families in this ward experience when accessing programs or support that maintain their health and wellbeing and that of their unborn children?
3. How are community members helping pregnant women and their families in this ward to access programs or support that maintain their health and wellbeing and that of their unborn children?
4. What are government and NGOs doing to enhance the support provided by relatives, friends, and the community in this ward to pregnant women and their families to access programs or support that maintain their health and wellbeing and that of their unborn children?
5. What are government and NGOs doing to enhance cultural norms and practices that enable communities in this ward to unite and support pregnant women and their families to access programs or support that maintain their health and wellbeing and that of their unborn children?
6. If you had the authority to do this, in what ways could you improve the support provided by relatives, friends, and the community in this ward that enables pregnant women and their families to access programs or support that maintain their health and wellbeing and that of their unborn children?
7. If you had the authority to do this, in what ways could you strengthen cultural norms and practices that enable people in this ward to unite and support pregnant women and their families to access programs or support that maintain their health and wellbeing and that of their unborn children?

Group presentations and final discussion

Now, each group will have the opportunity to share what they have discussed in their group. When all groups have finished, we will reflect on all the responses in our final discussion (*all groups take turns to share their responses, followed by a final discussion*).
